# Identifying incident colorectal and lung cancer cases in health service utilisation databases in Australia: a validation study

**DOI:** 10.1186/s12911-017-0417-5

**Published:** 2017-02-27

**Authors:** David Goldsbury, Marianne Weber, Sarsha Yap, Emily Banks, Dianne L. O’Connell, Karen Canfell

**Affiliations:** 1 Cancer Research Division, Cancer Council New South Wales, Sydney, Australia; 2 National Centre for Epidemiology and Population Health, Australian National University, Canberra, Australia; 3 Sydney School of Public Health, University of Sydney, Sydney, Australia; 4 School of Medicine and Public Health, University of Newcastle, Newcastle, Australia; 5 Prince of Wales Clinical School, UNSW, Sydney, Australia

**Keywords:** Colorectal cancer, Lung cancer, Case ascertainment, 45 and Up Study, Hospital diagnosis, Sensitivity, Specificity, Positive predictive value, Data linkage, Validation

## Abstract

**Background:**

Data from centralised, population-based statutory cancer registries are generally considered the 'gold standard' for confirming incident cases of cancer. When these are not available, or more current information is needed, hospital or other routinely collected population-level data may be feasible alternative sources. We aimed to determine the validity of various methods using routinely collected administrative health data for ascertaining incident cases of colorectal or lung cancer in participants from the 45 and Up Study in New South Wales (NSW), Australia.

**Methods:**

For 266,844 participants in the 45 and Up Study (recruited 2006–2009) ascertainment of incident colorectal or lung cancers was assessed using diagnosis and treatment records in linked administrative health datasets (hospital, emergency department, Medicare and pharmaceutical claims, death records). This was compared with ascertainment via the NSW Cancer Registry (NSWCR, the 'gold standard') for a period for which both data sources were available for participants.

**Results:**

A total of 2253 colorectal and 1019 lung cancers were recorded for study participants in the NSWCR over the period 2006–2010. A diagnosis of primary cancer recorded in the statewide Admitted Patient Data Collection identified the majority of NSWCR colorectal and lung cancers, with sensitivities and positive predictive values (PPV) of 95% and 91% for colorectal cancer and 81% and 85% for lung cancer, respectively. Using additional information on lung cancer deaths from death records increased sensitivity to 84% (PPV 83%) for lung cancer, but did not improve ascertainment of colorectal cancers. Hospital procedure codes for colorectal cancer surgery identified cases with sensitivity 81% and PPV 54%. No other individual indicator had sensitivity >50% or PPV >65% for either cancer type and no combination of indicators increased both the sensitivity and PPV above that achieved using the hospital cancer diagnosis data. All specificities were close to 100%; 95% confidence intervals for sensitivity and PPV were generally +/−2%.

**Conclusions:**

In NSW, identifying new cases of colorectal and lung cancer from administrative health datasets, such as hospital records, is a feasible alternative when cancer registry data are not available. However, the strengths and limitations of the different data sources should be borne in mind.

**Electronic supplementary material:**

The online version of this article (doi:10.1186/s12911-017-0417-5) contains supplementary material, which is available to authorized users.

## Background

Colorectal and lung cancer are two of the most common cancers, and the most frequent causes of cancer death in Australia [1]. The burden of these diseases is significant, both in terms of health expenditure and morbidity, not only in Australia but in almost all developed and developing countries [2]. Understanding and optimising the prevention, pathways to diagnosis and patterns of care for these two cancer types, as well as monitoring the effectiveness of cancer control initiatives is a national imperative [3].

Prospective linkage of cohort study questionnaire data to administrative, routinely collected population health datasets provides an effective method for identification of health outcomes, such as incident colorectal and lung cancers. This ascertainment of cancer cases allows for powerful and highly efficient investigation of the factors influencing cancer incidence, mortality, treatment and survival, which are important for research, evaluation and planning. Such linkage provides almost complete participant follow-up for health outcomes, without the cost, time, burden, and inaccuracies of self-report.

For cancer outcomes, the New South Wales Cancer Registry (NSWCR; a state-based statutory cancer registry) is the ‘gold standard’ for identifying cancer cases in NSW, Australia’s most populous state. The extensive ascertainment and quality assurance processes underpinning the NSWCR data mean that these data may not be released until several years after cancers are diagnosed, so study power may be reduced and contemporary analyses of factors influencing cancer diagnosis or outcomes may be more difficult to conduct. Identifying cancer cases via 'surrogate' indicators in other health databases, such as hospital records, which often are more contemporaneous, may provide a valid substitute for ascertaining certain types of incident cancers [4]. A large proportion of people with colorectal or lung cancer are hospitalised, either for treatment or because of medical complications, making it a potentially viable option to identify them in hospital records [5–7]. In a previous report, Kemp, et al. demonstrated high specificity and sensitivity for detecting invasive breast cancer using routinely reported hospital records in NSW [4]. However no previous study in Australia has investigated the use of this approach for any other cancer type.

Our objective was, therefore, to use a large-scale population-based Australian cohort study (the 45 and Up Study [8]) linked to a range of administrative health datasets including the NSWCR, to develop and investigate methods for identifying cases of colorectal and lung cancer up to the most recent date possible. Our specific aims were to use the 45 and Up Study cohort to: 1) devise algorithms for identifying incident cases of colorectal and lung cancer using non-NSWCR datasets; 2) validate these algorithms against the ‘gold standard’ NSWCR dataset from 2006 to 2010 (i.e. a time period in which complete data were available for both the NSWCR ‘gold standard’ and the other health datasets) to determine the optimal algorithm; and 3) identify new cases of colorectal and lung cancer in non-NSWCR datasets from 2011 onwards. Exploratory analyses examined the validity of hospital data for the ascertainment of other cancer types, including prostate cancer, breast cancer and melanoma.

## Methods

### Study sample

The Sax Institute’s 45 and Up Study in NSW, Australia, is a prospective cohort study of healthy ageing in which more than 266,000 participants consented for ongoing linkage of individual self-reported data to data from their medical records [8]. Eligible participants are men and women aged 45 years and over, randomly sampled from the Medicare enrolment database of the Department of Human Services (formerly Medicare Australia), which provides near complete coverage of the population. Individuals joined the study by completing a postal questionnaire (baseline questionnaires were distributed 2006–2009) and giving informed consent for linkage of their data to population health databases. People aged 80 years and over and those living in regional and remote areas were oversampled by a factor of 2. The study methods and a characterisation of the cohort are described in detail elsewhere [8, 9]. Participants aged less than 45 years at baseline and those with irreconcilable information in their linked records (e.g. multiple hospital admissions after date of death) were excluded.

Ethical approval for the 45 and Up Study as a whole was provided by the University of New South Wales Human Research Ethics Committee and specifically for this analysis by the NSW Population and Health Services Research Ethics Committee.

### Data sources and probabilistic record linkage

**Fig. 1:**
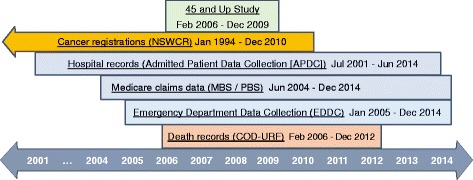
Health-related data collections used in the study and dates of available data. APDC: Admitted Patient Data Collection; COD-URF: Cause Of Death Unit Record File; EDDC: Emergency Department Data Collection; MBS: Medicare Benefits Schedule; NSWCR: New South Wales Cancer Registry; PBS: Pharmaceutical Benefits Scheme

Questionnaire data from study participants were linked probabilistically to a number of population-wide health databases (Fig. 1): (1) NSW Admitted Patient Data Collection (APDC; July 2001–June 2014), which is a complete census of all public and private hospital admissions in NSW. Hospital medical coders abstract individual patient information from medical records following the patient’s discharge from hospital. This includes dates of admission and separation, procedures carried out and diagnoses relating to the hospital episode; (2) Emergency Department Data Collection (EDDC; January 2005–December 2014), which records presentations to emergency departments in most public hospitals across NSW; (3) NSW Cancer Registry (NSWCR; January 1994–December 2010), which records details of all notifications of primary cancer diagnoses for residents of NSW; (4) Australian Coordinating Registry Cause of Death Unit Record File (COD-URF; January 2006–December 2012), which contains information about causes of death in Australia, taken from death records.

These datasets were probabilistically linked by the Centre for Health Record Linkage [10] using a best practice approach to linkage while preserving privacy [11] and the open source probabilistic record linkage software ChoiceMaker. The probabilistic matching process is known to be highly accurate (false-positive and false-negative rates <0.5%) and a more detailed description of the linkage process has been provided elsewhere [12]. Medication use and subsidised outpatient and medical services from June 2004 to December 2014 were available for all study participants from the Department of Human Services via linkage to the Pharmaceutical Benefits Scheme (PBS; a database of all government subsidised prescription pharmaceuticals) and the Medicare Benefits Schedule (MBS; a database of the Medicare services subsidised by the Australian government and available to all Australian residents) using a unique identifier that was provided to the Department of Human Services by the Sax Institute.

### Ascertainment of cancer cases in the NSWCR

Colorectal and lung cancers were defined as primary invasive cancers that were diagnosed according to the NSWCR between January 1994 and December 2010. Using the 10th revision of the International Classification of Disease, Australian Modification (ICD10-AM), colorectal cancers were coded as C18-C20 and lung cancers as C34. Month and year of diagnosis were supplied, but not the day of diagnosis.

### Ascertainment of cancer cases in other health datasets

The possible indicators for identifying new cases of colorectal and lung cancer in each dataset are listed in Table 1. Hospital discharge diagnosis data from the APDC comprised up to 55 diagnosis fields for each admission, coded according to ICD10-AM. The diagnosis information included the reason for the hospital admission or factors relating to the hospital stay, which could include a new/recent diagnosis of cancer (referred to here as a “hospital cancer diagnosis record”, using codes C18-C20 for colorectal cancer and C34 for lung cancer) or a record indicating a personal history of the disease (not a new/recent diagnosis of cancer, using code Z85.0 for colorectal cancer and Z85.1 for lung cancer). Secondary colorectal or lung cancers, such as brain cancer that had metastasised to the lungs, were not included in this analysis. EDDC records had one diagnosis for each presentation, coded according to either the ICD10-AM, ICD 9th revision (ICD9) or SNOMED classification system.

**Table 1:** Validity of potential indicators for lung and colorectal cancer compared with the NSW Cancer Registry, February 2006 to December 2010

Source	Colorectal cancer (No. cases in NSWCR: 2253)				Lung cancer (No. cases in NSWCR: 1019)			
	No. cases identified	PPV	Sensitivity	Specificity	No. cases identified	PPV	Sensitivity	Specificity
Individual indicators								
APDC diagnosis of cancer	2338	91%	95%	99.9%	968	85%	81%	100.0%
APDC diagnosis of history of primary cancer	2487	24%	26%	99.5%	166	23%	4%	100.0%
APDC surgical resection	3376	54%	81%	99.4%	400	60%	23%	99.9%
APDC chemotherapy	1333	12%	7%	99.6%	1333	6%	7%	99.5%
APDC radiotherapy	456	4%	1%	99.8%	456	14%	6%	99.9%
EDDC diagnosis of primary cancer	27	44%	1%	100.0%	56	50%	3%	100.0%
MBS surgical resection	1793	59%	47%	99.7%	310	45%	14%	99.9%
MBS radiotherapy item	6051	3%	9%	97.8%	6051	5%	33%	97.9%
MBS chemotherapy item	4940	12%	27%	98.4%	4940	7%	35%	98.3%
PBS chemotherapy drug item	1663	46%	34%	99.7%	2082	14%	28%	99.3%
COD-URF death record with primary cancer type	364	36%	6%	99.9%	609	62%	37%	100.0%
Combinations of indicators								
APDC cancer diagnosis or history of cancer	3254	65%	94%	99.6%	1016	81%	81%	99.9%
APDC cancer diagnosis or surgery	3738	57%	95%	99.4%	1119	74%	81%	99.9%
APDC cancer diagnosis and surgery	1933	94%	80%	100.0%	246	96%	23%	100.0%
APDC cancer diagnosis and APDC chemotherapy or radiotherapy	201	83%	7%	100.0%	145	83%	12%	100.0%
APDC or EDDC diagnosis of primary cancer	2345	91%	95%	99.9%	980	84%	81%	100.0%
MBS or PBS chemotherapy or radiotherapy	9144	9%	38%	96.9%	9178	5%	49%	96.8%
APDC cancer diagnosis and MBS/PBS chemotherapy or radiotherapy	963	86%	37%	100.0%	526	79%	41%	100.0%
APDC cancer diagnosis and COD-URF primary cancer	242	52%	6%	100.0%	483	71%	34%	100.0%
APDC cancer diagnosis or COD-URF primary cancer	2370	90%	95%	99.9%	1042	83%	84%	99.9%

*APDC* Admitted Patient Data Collection, *COD-URF* Cause Of Death Unit Record File, *EDDC* Emergency Department Data Collection, *MBS* Medicare Benefits Schedule, *NSWCR* New South Wales Cancer Registry, *PBS* Pharmaceutical Benefits Scheme, *PPV* Positive predictive value

Colorectal and lung cancer treatments were captured in the APDC where up to 50 procedure codes could be recorded at each admission and by a single item code per claim in the MBS (see Additional file 1 for details of diagnosis, procedure and item codes used). Procedures in the APDC were coded using the Australian Classification of Health Interventions, which is used in conjunction with ICD10-AM. Chemotherapy medicines listed on the PBS for colorectal cancer included fluorouracil and oxaliplatin, while for lung cancer the medicines included carboplatin, cisplatin and docetaxel (see Additional file 1 for details). The included treatments were indicated for colorectal and lung cancers but were not necessarily exclusive to these conditions. The COD-URF was used to identify deaths with primary colorectal or lung cancer (not secondary colorectal or lung cancer) as the main cause of death or one of up to 20 contributing causes of death.

### Statistical methods

Sensitivity, specificity and the positive predictive value (PPV) of ascertaining cases of cancer for February 2006 to December 2010 (the period when data were available for all datasets) in non-NSWCR datasets compared to the ‘gold standard’ NSWCR were calculated separately for colorectal and lung cancer. A true positive was defined as a colorectal/lung cancer that was first identified in a non-NSWCR dataset up to 12 months before or after a NSWCR-recorded colorectal/lung cancer diagnosis date, where the date for the non-NSWCR diagnosis was the first date of its occurrence in the dataset (e.g. hospital admission date as opposed to the discharge date, ED presentation date or service/supply date). A true negative was defined as the absence of a derived indicator of colorectal/lung cancer in a non-NSWCR dataset and the absence of a colorectal/lung cancer record in the NSWCR. Sensitivity, specificity and PPV were calculated using the NSWCR as the reference standard. Sensitivity was calculated as the proportion of all cases in the NSWCR who were true positives in the relevant non-NSWCR dataset. Specificity was calculated as the proportion of all people who were not identified as cases in the NSWCR who were true negatives in the relevant non-NSWCR dataset. PPV was defined as the proportion of all cases identified in the relevant non-NSWCR dataset who were true positives in that non-NSWCR dataset.

Sensitivity, specificity and PPV were compared for algorithms using different combinations of colorectal/lung cancer indicators from non-NSWCR datasets to determine the optimal algorithm for case ascertainment for each cancer type. The main focus of the paper was estimating sensitivity, specificity and PPV based on true positives being within +/− 12 months, however we also estimated these measures based on true positives being within +/− 3 months. After identifying the optimal algorithm we also assessed the sensitivity, specificity and PPV for different follow-up periods, using data prior to 2006 where available. Using the optimal algorithm, colorectal and lung cancer cases were then identified in non-NSWCR datasets beyond 2010 to determine how use of the algorithm might impact the number of cases (and hence statistical power) for further analysis of risk factors, health services utilisation, and cancer outcomes. In order to investigate whether there were any systematic biases underpinning the ‘missed’ and ‘extra’ incident cancer cases in the hospital records, we further assessed these cases in relation to their cancer stage, area-level socio-economic disadvantage, geographic area of residence and vital status and timing of death. Preliminary testing was also carried out for other cancer types, using the most common incident (post-baseline) cancer types in the NSWCR for study participants (Table 2).

**Table 2:** Validity of APDC cancer diagnoses compared with the NSW Cancer Registry for selected cancer types, July 2001 to December 2010

Cancer type	Cases in NSWCR	Cases in APDC	PPV	Sensitivity	Specificity
Bladder	523	1253	35%	84%	99.7%
Breast	4172	4376	86%	90%	99.8%
Colorectal	3597	3747	91%	95%	99.9%
Kidney	575	616	85%	91%	100.0%
Lung	1225	1151	86%	81%	100.0%
Melanoma	3748	2459	71%	47%	99.8%
Non-Hodgkin Lymphoma	1003	1080	71%	76%	99.9%
Pancreatic	271	283	81%	84%	100.0%
Prostate	7256	6401	86%	76%	99.7%
Stomach	282	314	78%	87%	100.0%
Unknown primary	306	659	29%	62%	99.8%
Uterine	543	535	93%	92%	100.0%

*APDC* Admitted Patient Data Collection, *NSWCR* New South Wales Cancer Registry, *PPV* Positive predictive value

## Results

There were 266,844 participants recruited to the 45 and Up Study in 2006–2009. We excluded 50 people (0.02%) who were either aged less than 45 years at baseline or who had linked health-related data that could not be reconciled (e.g. multiple hospital admissions after date of death), leaving 266,794 eligible participants.

There were 2253 people with a diagnosis of colorectal cancer and 1019 with lung cancer recorded in the NSWCR over the period February 2006–December 2010. This is the period when data were available from the NSWCR and all of the other administrative data collections (Fig. 1). These cancers were newly diagnosed during that period, but were not necessarily diagnosed after the baseline questionnaire.

### Comparison of potential surrogate indicators for cancer

Table 1 shows the validity of individual indicators for colorectal and lung cancers in the non-NSWCR datasets. Having a hospital diagnosis code for the primary cancer type (colorectal or lung) was by far the best individual indicator for both cancer types, with sensitivity and PPV of 95% and 91% respectively for colorectal cancer and 81% and 85% respectively for lung cancer. The 95% confidence intervals for these measures were within +/− 1.2% for colorectal cancer and within +/− 2.4% for lung cancer. Hospital records for colorectal cancer surgical procedures identified cases with 81% sensitivity and 54% PPV, but no other individual marker had >50% sensitivity or >65% PPV for colorectal or lung cancer (Table 1). The specificities for each of the individual indicators and combinations of indicators were at least 98%, with most being >99.8% and all 95% confidence intervals for specificity were at least 96% to at most 100%. The results for hospital cancer diagnosis records were very similar when the NSWCR cancers were restricted to those diagnosed after the baseline questionnaire.

Sensitivity for lung cancer increased from 81% using a hospital cancer diagnosis, to 84% using the combination of a hospital cancer diagnosis and/or a death record where lung cancer was recorded as a cause of death. However the PPV decreased slightly from 85% (hospital cancer diagnosis only) to 83% (hospital cancer diagnosis and/or death record). The inclusion of death records for colorectal cancer did not improve identification of colorectal cancers, nor did any combination of indicators increase both the sensitivity and PPV above that obtained using the hospital cancer diagnosis for either cancer type (Table 1). Some combinations, for example a hospital cancer diagnosis plus a surgical resection record, had slightly higher PPV (94% for colorectal cancer) but substantially lower sensitivity (80%). The death records were available to December 2012, compared to June 2014 for the hospital records, so using the combination of hospital and death records beyond the NSWCR coverage would also introduce complications due to differing data availability. Based on these results, we considered the hospital cancer diagnosis records to be the optimal ‘surrogate’ for identifying new colorectal and lung cancers. The remaining analysis focused on this data source.

### Indicators based on hospital cancer diagnoses

**Fig. 2:**
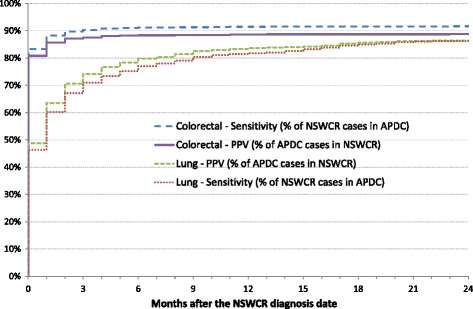
Sensitivity and PPV of hospital diagnosis records after the NSW Cancer Registry diagnosis date, February 2006 to December 2010. APDC: Admitted Patient Data Collection; NSWCR: New South Wales Cancer Registry; PPV: Positive predictive value. Does not include cancers identified from the APDC prior to the NSWCR diagnosis date (3% of colorectal cancers, 2% of lung cancers)

When true positive cases were restricted to those with a hospital cancer diagnosis up to 3 months before or after the NSWCR diagnosis date, the sensitivity and PPV of a hospital colorectal cancer diagnosis both decreased by 1% to 94% and 90% respectively. For lung cancer, the sensitivity and PPV of a hospital cancer diagnosis dropped from 81% to 72% and from 85% to 76% respectively. Hospital cancer diagnoses first identified 83% of the NSWCR colorectal cancers and 47% of lung cancers in the same month as the NSWCR diagnosis date. For both cancer types around 2–3% of cases were first identified from a hospital admission in the month prior to the month of diagnosis in the NSWCR, with very few cases (<5) identified earlier than 1 month prior to the NSWCR diagnosis date. As shown in Fig. 2, the majority of cancers were identified in the same month or the initial months after the NSWCR diagnosis date (the 2–3% in the month prior to the NSWCR date are not shown).

Restricting the comparison to the NSWCR and hospital cancer diagnosis records, there was a 9.5-year period of overlap between the datasets where we could compare diagnoses (July 2001–December 2010). Defining true positive cases as those up to 12 months before or after the NSWCR diagnosis date, the results were very similar to those for the period February 2006–December 2010, with sensitivity and PPV of 95% and 91% respectively for colorectal cancer (3597 NSWCR cases) and 81% and 86% for lung cancer (1225 NSWCR cases) (Table 2).

### Further investigation of ‘missed’ and ‘extra’ cancer diagnoses in the inpatient hospital data

Of the 3597 colorectal cancer cases in the NSWCR, the APDC did not identify 195 (5%) within 12 months of the NSWCR diagnosis date. Of these, 24 were identified in the APDC after >12 months or after December 2010 (to June 2014), while seven died from colorectal cancer within 12 months. Of the remaining 136 cases, around half lived in areas near the border of another state or territory, so they may have been hospitalised interstate and these are not captured in the study datasets. The NSWCR cases who were not identified in the APDC more commonly had “unknown” spread of disease at diagnosis recorded in the NSWCR (33% vs 7% of those who were in the APDC) but the proportions with distant metastases recorded were similar (9% vs 10%) and there was little difference by age at diagnosis in the NSWCR (median 68 vs 69). The APDC also identified 332 colorectal cancer cases who were not in the NSWCR during July 2001–December 2010. Of these, 123 (37%) were recorded in the NSWCR prior to July 2001 (from January 1994). Comparing cases identified in the APDC over the 9.5-year period to all cases recorded in the NSWCR without considering the dates of these records, the APDC had a PPV of 94%.

The sensitivity with which the hospital cancer diagnoses identified NSWCR lung cancer cases for the period July 2001 to December 2010 (81%) increased to 85% if we included cases identified in the APDC >12 months after the date of diagnosis in the NSWCR. If we also included APDC cases first identified after December 2010, the sensitivity increased to 91%. Lung cancer death records identified a further 4% of the NSWCR cases. The PPV (86%) increased to 90% if we included cases identified in the APDC >12 months after the date of diagnosis in the NSWCR and then to 93% if we included NSWCR cases diagnosed prior to July 2001.

Comparisons were made between the NSWCR lung cancer cases who were identified in the APDC within 12 months of the NSWCR date of diagnosis (“matches”, *n* = 991, 81%) and those who were not (“non-matches”, *n* = 234, 19%). The non-matches were more likely to have “unknown” spread of disease at diagnosis recorded in the NSWCR (45% of non-matches vs 13% of matches), were more commonly from non-metropolitan areas (56% of non-matches vs 45% of matches) or lower socio-economic areas (60% of non-matches were from the two most disadvantaged quintiles vs 50% of matches) and were slightly older at diagnosis according to the NSWCR (median 73 years [inter-quartile range 65–81] for non-matches vs 71 [63–79] for matches). The non-matches were more commonly from areas near the border of another state or territory (21% vs 8% of matches), so they may have been hospitalised interstate and these are not captured in the study datasets.

Of the 234 non-matches, 19 (8%) died within 1 month of the date of diagnosis recorded in the NSWCR (16 were identified using lung cancer death records) and of these, 13 did not have a hospital admission during or after the month of diagnosis. The inclusion of lung cancer death records increased the sensitivity of identification by 3% but reduced the PPV by 2%. This reduction was due to 37 people having a lung cancer death record during July 2001–December 2010 but who did not have a lung cancer diagnosis recorded in the NSWCR during that time. Based on all of the available data for these 37 people, 10 had a diagnosis of lung cancer recorded in the NSWCR prior to July 2001 or data suggesting they may have had lung cancer that was not captured in the available NSWCR dataset (e.g. pre-1994 or false-negative linkage), 13 were potentially secondary lung cancers and 14 may have had some other form of lung disease.

### Impact of using inpatient hospital data on the numbers of incident cases available for other analyses

There were 1319 participants with incident colorectal cancer (diagnosed after joining the study) recorded in the NSWCR to December 2010. Using cases first identified by an APDC diagnosis after December 2010 added 1549 colorectal cancer cases up to June 2014, an increase of 117%.

There were 795 participants with incident lung cancer recorded in the NSWCR to December 2010 and the APDC diagnoses added a further 912 cancer cases to June 2014, an increase of 115%. Also including cases identified from cause of death on death records added a further 51 lung cancer cases, although COD-URF records were only available to December 2012.

### Exploratory analysis for other cancer types

Given our finding that the diagnosis codes in the APDC were the optimal ‘surrogate’ method for identifying colorectal and lung cancer types, we also compared cancer diagnoses in the APDC with NSWCR records for several other common cancer types (Table 2). The results show substantial variation by cancer type, with sensitivities ranging from 47% for melanoma to 92% for uterine cancer. Lower sensitivities were observed for cancers where there is less inpatient treatment required (e.g. melanoma 47%, prostate 76%) and lower PPV for cancer of unknown primary (29%) where there is less certainty about the cancer type – 81% of unknown primary cases according to the APDC also had another primary cancer type recorded in the APDC. There was also lower PPV for bladder cancer (35%), where many cases have long survival and regular inpatient follow-up (e.g. cystoscopy), so the hospital cancer diagnosis was identifying a cancer diagnosed several years earlier and not a new case, or it might be an *in situ* or non-invasive case as these are not recorded in the NSWCR [13]. For some cancer types where the sensitivity was lower there may be other health-related records that could help identify cases, such as Medicare records of outpatient excision biopsies for localised melanoma cases, but a detailed investigation of the relevant data sources for this and the other cancer types is beyond the scope of the current analysis.

## Discussion

In this analysis of more than 266,000 individuals with follow-up of almost 5 years, we found that hospital diagnoses of colorectal cancer can be used to reliably identify and/or rule out incident cases in the absence of cancer registry data. Hospital diagnoses of lung cancer were not as comprehensive or timely as those for colorectal cancer, but still provide a reasonable indicator for incident lung cancers. However, ascertainment of lung cancer diagnosis can be improved via the use of lung cancer death records.

Our results for lung and colorectal cancer broadly concur with a previous study of breast cancer in 45 and Up Study participants. Kemp et al. reported that APDC diagnosis codes identified incident breast cancers with 86% sensitivity and 86% PPV in 2004–2008 [4]. In our analysis of breast cancer, PPV was 86% and sensitivity was 90%. Restricting to female cases diagnosed in 2004–2008 made no material difference to our results, which are comparable to the findings of Kemp et al. The improved sensitivity in our analysis can be explained because Kemp et al. used a 3 month window for identification of true positives and only included the principal diagnosis code at each hospital admission, whereas we used a 12 month window and included all diagnoses recorded at each admission. The higher sensitivity and unchanged PPV suggests that the latter is the optimal algorithm. In our study, we have built on the work of Kemp et al. to show that other common cancer types (colorectal, lung) are also amenable to the use of surrogate outcome markers of diagnosis in routinely collected hospital data. However, we have also shown in exploratory analysis that not all cancer types are necessarily amenable to this approach, especially if treatment does not routinely occur on an in-patient basis.

### Colorectal cancers

We found that there was very high validity for hospital diagnosis records for identifying colorectal cancer, which is likely to be driven by the high surgery/treatment rates within a short period after diagnosis for this cancer type. In contrast, previous studies have found that around one-third of lung cancer cases in NSW do not receive cancer-specific treatment [14] so there are fewer opportunities to be identified with a diagnosis of lung cancer in hospital records. Nevertheless, many patients with lung cancer are hospitalised for complications from outpatient services such as radiotherapy, and complications from lung cancer itself, which may explain the >80% sensitivity based on a diagnosis recorded in the APDC in our study.

### Lung cancers

We found that ascertainment of lung cancer diagnosis can be improved via the use of lung cancer death records for this low survival disease. It should, however, be borne in mind that such a strategy will preferentially identify fatal lung cancers over non-fatal cancers, so this may not be an appropriate ‘surrogate’ diagnostic marker for lung cancer for all analyses. The inclusion of death records improved the sensitivity with which lung cancer was identified, but led to a slight reduction in PPV. The death records often identified people who died shortly after being diagnosed and who did not have a long period of time to use health services and therefore had less chance of being identified in the hospital data. The reduction in PPV caused by the inclusion of death records was partly due to the introduction of people who died from cancer but who were diagnosed in the NSWCR before the study period, along with the inclusion of people who most likely had secondary lung cancer metastasised from another primary site or who had other lung disease but were classified as having primary lung cancer on their death certificate.

### Limitations

One limitation of our analysis is that although it is population-based, the 45 and Up Study is not strictly representative of the general population [9], with those in marginalised groups less likely to participate in studies of this type. For that reason, hospital data might not be as sensitive for population-wide identification of lung cancer as for other cancer types, given that socio-economic differences exist in smoking rates and in patterns of lung cancer care [6]. However, using an available dataset containing all NSWCR records for 2001–2009 and their hospital records for 2000–2011, we ran the same analysis using hospital diagnosis records for the whole NSW population and obtained similar estimates for sensitivity for colorectal and lung cancers (data not shown). Also, the results reflect the data for the study period and might not be representative of later time periods. The results also included cancers diagnosed prior to entry into the 45 and Up Study and so might not be representative of future incident cancers, but the results were very similar when only post-baseline cancers were included. The narrow confidence intervals for sensitivity, PPV and specificity due to the very large cohort and numbers of cases are strengths of the study, but it is also a reflection of the precision of the estimates and not necessarily their accuracy, as other statistical uncertainty cannot be excluded.

There are also some limitations to the suggested surrogate markers for incident cancers. The hospital data do not include information about disease stage or the actual date of diagnosis, which are often important data items required for cancer-related studies such as assessing the appropriateness or timeliness of treatment. In NSW, pathology is performed through a mix of private and public hospital laboratories, with no one pathology database covering the entire population, so detailed individual-level pathology data beyond those included in cancer registry data were not available. We also did some preliminary investigating of cancer site/location recorded in the hospital records (data not shown) and they often varied and were different to those recorded in the cancer registry data, such as the recording of rectosigmoid cancer as rectal cancer or vice versa. However, hospital data can be used to identify important health-related information that is not available from cancer registries, such as the presence of various comorbid conditions over time.

The surrogate indicators for cancer, particularly for lung cancer, tended to lag behind the actual diagnosis date, although around three-quarters were identified up to 3 months before or after the NSWCR recorded date of diagnosis. Using these sources would result in a small dip in the number of cases in the months after the cancer registry data ends, due to the surrogate indicators identifying cases already covered by the final cancer registry data, but it would then return to around the expected level. The time lag might be important if trying to assess a relatively short time-related factor such as the timeliness of treatment after diagnosis or short-term survival, but for overall incidence it is a reasonable measure. There were cases who were not identified using the hospital records, in particular for lung cancer. Using hospital records alone will miss a small proportion of new cases, and they tend to be the people with less health system contact, such as those with unknown disease stage or from non-metropolitan areas. This also suggests that if hospital data are used to calculate incidence, they will give a slight underestimate and could attenuate differences between cancer cases and non-cases in analyses of risk factors for cancer. However this relates to a relatively small proportion of the overall number of cases. The criteria used to assess the validity of the surrogate indicator algorithms are not perfect, particularly for people diagnosed at the start/end of the study period. For example, someone diagnosed in the NSWCR in December 2010 and in the APDC in January 2011 would be considered a ‘false negative’ in the APDC due to the study period date cut-off. However we believe that overall the criteria used provide a strong and objective measure of validity for comparisons.

Furthermore, there are some limitations relating to the study we have undertaken. The primary purposes of the non-cancer registry data sources do not include cancer identification or recording, so they should be used for this purpose with caution. It is also possible that the collection of hospital data might change in future and this could impact upon their validity for identifying incident cancers. There is a small chance of false negative or false positive linkage, which can have an impact when there is a relatively small proportion of cases. Finally, the NSWCR data for 2011 and COD-URF data for 2013 became available as we were completing this study (in 2016), but we have not yet gained access to these data to allow for further analysis.

In this analysis of specific cancer types, we found that an algorithm based on hospital records, rather than emergency department records or Medicare claims, was the most accessible, practical and valid method for ascertaining cancer diagnosis. The EDDC is a rich and useful dataset in its own right, but it does not appear to contribute to the identification of cancer cases. The EDDC data custodian warns against the use of diagnosis fields in the EDDC for analytical purposes, as only one diagnosis is recorded per presentation and it is not coded consistently across all EDs in the state [10]. Furthermore, the EDDC did not capture all EDs in the state throughout the study period. It covered around 80% of ED presentations in 2007, with coverage continuing to steadily increase since 2005 [10]. Despite these limitations, the EDDC still provides powerful information about an important part of patient care. Similarly there was a great deal of information gained from the claims records in the MBS and PBS. The data identified many thousands of people who had cancer treatment and provide an excellent insight into patient care, but the recorded items may not be specific to cancer types (e.g. chemotherapy medicines such as docetaxel can be used for several different cancer types) so by themselves they may not be useful as surrogate indicators for these specific cancers. Future work, however, will explore methods for overcoming such issues via the use of probabilistic algorithmic approaches using the rich information in all of the available datasets.

### Implications

For ongoing cohort studies there is great benefit in having cancer incidence data that are as current as possible, allowing for more timely and relevant examination of cancer-related outcomes, as well as a greater number of cases to increase the power to detect associations. Furthermore, for countries that lack centralised cancer registries, being able to estimate cancer incidence through hospital and other medical records is of benefit for research, surveillance and planning purposes.

APDC diagnosis records for colorectal or lung cancer were adequate for identifying new cases of these cancer types in this prospective cohort study. Using the APDC to identify new cases of colorectal and lung cancer provides more up-to-date cancer incidence data and permits investigation of a range of topics with greater follow-up time from entry into the study and higher statistical power. Using the APDC diagnosis data to the end of the follow-up period (June 2014) more than doubled the number of incident cancers since entry into the study compared to using cancer registry data alone, increasing the median follow-up time from 2.5 to 6 years and providing greater power to, for example, detect associations between risk factors and cancer incidence. Using hospital records, the vast majority of cases in the cohort were picked up and those who were identified as having cancer are highly likely to be true cases. The extremely large cohort provided large numbers of cases and precise estimates and the use of population-based datasets allows for excellent coverage of the cohort and the conditions of interest.

## Conclusions

In conclusion, identifying cases of colorectal and lung cancer in administrative health datasets, such as hospital records, may be a feasible alternative to use of cancer registry data in Australia, provided that the limitations of such data are carefully borne in mind. We found that a hospital inpatient diagnosis of colorectal or lung cancer is the most valid individual surrogate indicator for these cancer types and is relatively simple to implement.

## Additional file

Additional file 1: Codes to potentially identify colorectal and lung cancers. (DOCX 40 kb)

## Abbreviations

APDC: Admitted Patient Data Collection
COD-URF: Cause of Death Unit Record File
ED: Emergency Department
EDDC: Emergency Department Data Collection
ICD: International Classification of Disease
MBS: Medicare Benefits Schedule
NSW: New South Wales
NSWCR: New South Wales Cancer Registry
PBS: Pharmaceutical Benefits Scheme
PPV: Positive predictive value
