# Supplementary material for: Identifying incident colorectal and lung cancer cases in health service utilisation databases in Australia: a validation study
Source: BMC Med Inform Decis Mak. 2017 Feb 27;17:23. doi: 10.1186/s12911-017-0417-5 (PMC5327557; doi:10.1186/s12911-017-0417-5)
Supplement: Additional file 1: — Codes to potentially identify colorectal and lung cancers. (DOCX 40 kb) [file 12911_2017_417_MOESM1_ESM.docx]

**Additional file 1**

**List of abbreviations**

APDC: Admitted Patient Data Collection

EDDC: Emergency Department Data Collection

ICD10-AM: International Classification of Disease, 10^th^ edition, Australian modification

ICD9: International Classification of Disease, 9^th^ edition

MBS: Medicare Benefits Schedule

PBS: Pharmaceutical Benefits Scheme

**Codes to potentially identify colorectal cancers**

Table S1. APDC procedure codes to identify surgical resection for colorectal cancer.

| **Code** | **Description** |
| --- | --- |
| 32000-00 | Limited excision of large intestine with formation of stoma |
| 32000-01 | Right hemicolectomy with formation of stoma |
| 32000-02 | Laparoscopic limited excision of large intestine with formation of stoma |
| 32000-03 | Laparoscopic right hemicolectomy with formation of stoma |
| 32003-00 | Limited excision of large intestine with anastomosis |
| 32003-01 | Right hemicolectomy with anastomosis |
| 32003-02 | Laparoscopic limited excision of large intestine with anastomosis |
| 32003-03 | Laparoscopic right hemicolectomy with anastomosis |
| 32004-00 | Subtotal colectomy with formation of stoma |
| 32004-01 | Extended right hemicolectomy with formation of stoma |
| 32004-02 | Laparoscopic subtotal colectomy with formation of stoma |
| 32004-03 | Laparoscopic extended right hemicolectomy with formation of stoma |
| 32005-00 | Subtotal colectomy with anastomosis |
| 32005-01 | Extended right hemicolectomy with anastomosis |
| 32005-02 | Laparoscopic subtotal colectomy with anastomosis |
| 32005-03 | Laparoscopic extended right hemicolectomy with anastomosis |
| 32006-00 | Left hemicolectomy with anastomosis |
| 32006-01 | Left hemicolectomy with formation of stoma |
| 32006-02 | Laparoscopic left hemicolectomy with anastomosis |
| 32006-03 | Laparoscopic left hemicolectomy with formation of stoma |
| 32009-00 | Total colectomy with ileostomy |
| 32009-01 | Laparoscopic total colectomy with ileostomy |
| 32012-00 | Total colectomy with ileorectal anastomosis |
| 32012-01 | Laparoscopic total colectomy with ileorectal anastomosis |
| 32015-00 | Total proctocolectomy with ileostomy |
| 32024-00 | High anterior resection of rectum |
| 32025-00 | Low anterior resection of rectum |
| 32026-00 | Ultra low anterior resection of rectum |
| 32028-00 | Ultra low anterior resection of rectum with hand sutured coloanal anastomosis |
| 32030-00 | Rectosigmoidectomy with formation of stoma (Hartmann’s procedure) |
| 32030-01 | Laparoscopic rectosigmoidectomy with formation of stoma |
| 32033-00 | Restoration of bowel continuity after Hartmann's procedure |
| 32039-00 | Abdominoperineal proctectomy |
| 32047-00 | Perineal proctectomy |
| 32051-00 | Total proctocolectomy with ileo-anal anastomosis |
| 32051-01 | Total proctocolectomy with ileo-anal anastomosis and formation of temporary ileostomy |
| 32051-03 | Total proctocolectomy with ileorectal anastomosis and formation of temporary ileostomy |
| 32099-00 | Per anal submucosal excision of lesion or tissue of rectum |
| 32105-00 | Per anal full thickness excision of anorectal lesion or tissue |
| 32108-00 | Transsphincteric excision of lesion or tissue of rectum |
| 32112-00 | Perineal rectosigmoidectomy |
| 90308-00 | Endoscopic destruction of lesion of large intestine |
| 90315-00 | Endoscopic excision of lesion or tissue of anus |
| 90315-01 | Excision of other lesion or tissue of anus |
| 90341-00 | Other excision of lesion of rectum |
| 90450-00 | Anterior pelvic exenteration |
| 90450-01 | Posterior pelvic exenteration |
| 90450-02 | Total pelvic exenteration |
| 90959-00 | Excision of other lesion of large intestine |

Table S2. APDC procedure codes and diagnosis codes to identify chemotherapy for colorectal cancer.

| **Data type** | **Code** | **Description** |
| --- | --- | --- |
| Procedure | 13915-00 | Chemotherapy, intravenous administration <= 1 hours duration |
| Procedure | 13918-00 | Chemotherapy, intravenous administration >1 - 6 hours duration |
| Procedure | 13921-00 | Chemotherapy, intravenous administration >6 hours duration |
| Procedure | 90760-00 | Chemotherapy, oral administration |
| Procedure | 90767-00 | Chemotherapy, subcutaneous or intramuscular administration |
| Procedure | 90768-00 | Chemotherapy, other administration |
| Diagnosis | Z51.1 | Pharmacotherapy session for neoplasm |
| Diagnosis | Z51.2 | Other chemotherapy |

Table S3. APDC procedure codes and diagnosis codes to identify radiotherapy for colorectal cancer.

| **Data type** | **Code** | **Description** |
| --- | --- | --- |
| Procedure | 15203-00 | Radiation treatment, megavoltage, single photon energy machine, 1 field |
| Procedure | 15204-00 | Radiation treatment, megavoltage, single photon energy machine, >= 2 fields |
| Procedure | 15207-00 | Radiation treatment, megavoltage, dual photon energy machine, 1 field |
| Procedure | 15208-00 | Radiation treatment, megavoltage, dual photon energy machine, >= 2 fields |
| Procedure | 15224-00 | Radiation treatment, megavoltage, single modality linear accelerator, 1 field |
| Procedure | 15239-00 | Radiation treatment, megavoltage, single modality linear accelerator, >= 2 fields |
| Procedure | 15254-00 | Radiation treatment, megavoltage, dual modality linear accelerator, 1 field |
| Procedure | 15269-00 | Radiation treatment, megavoltage, dual modality linear accelerator, >= 2 fields |
| Procedure | 15506-01 | Radiation field setting using dedicated CT scanner |
| Procedure | 15506-02 | Radiation field setting using intensity modulated radiation therapy |
| Procedure | 15521-00 | Dosimetry by CT interfacing computer, intermediate |
| Procedure | 15550-00 | Radiation field setting for three dimensional conformal radiation therapy |
| Procedure | 15600-00 | Stereotactic radiation treatment, single dose |
| Procedure | 15600-01 | Stereotactic radiation treatment, fractionated |
| Procedure | 90765-00 | Construction and fitting of immobilisation device, simple |
| Procedure | 90765-01 | Construction and fitting of immobilisation device, intermediate |
| Procedure | 90765-02 | Construction and fitting of immobilisation device, complex |
| Procedure | 90765-03 | Construction and fitting of customised blocks |
| Diagnosis | Z51.0 | Radiotherapy session |

Table S4. EDDC diagnosis codes to identify colorectal cancer.

| **Classification system** | **Code** | **Description** |
| --- | --- | --- |
| ICD10-AM | C18 | Malignant neoplasm of colon |
| ICD10-AM | C19 | Malignant neoplasm of rectosigmoid junction |
| ICD10-AM | C20 | Malignant neoplasm of rectum |
| ICD9 | 153 | Malignant neoplasm of colon |
| ICD9 | 154.0 | Malignant neoplasm of rectum, rectosigmoid junction and anus - Rectosigmoid junction |
| ICD9 | 154.1 | Malignant neoplasm of rectum, rectosigmoid junction and anus - Rectum |
| SNOMED | 126838000 | Neoplasm of colon (disorder) |
| SNOMED | 126845000 | Neoplasm of sigmoid colon (disorder) |

Table S5. MBS item codes to identify surgical resection for colorectal cancer.

| **Code** | **Description** |
| --- | --- |
| 20848 | Initiation of management of anaesthesia for pelvic exenteration |
| 32000 | Resection of large intestine, without anastomosis, including right hemicolectomy (including formation of stoma) |
| 32003 | Resection of large intestine, with anastomosis, including right hemicolectomy |
| 32004 | Subtotal colectomy of large intestine (resection of right colon, transverse colon & splenic flexure) without anastomosis |
| 32005 | Subtotal colectomy of large intestine (resection of right colon, transverse colon & splenic flexure) with anastomosis |
| 32006 | Left hemicolectomy, including the descending and sigmoid colon (including formation of stoma) |
| 32009 | Total colectomy and ileostomy |
| 32012 | Total colectomy and ileorectal anastomosis |
| 32015 | Total colectomy with excision of rectum and ileostomy, 1 surgeon |
| 32024 | High restorative anterior resection of rectum, with intraperitoneal anastomosis (of the rectum) >10cm from the anal verge, excluding resection of sigmoid colon alone |
| 32025 | Low restorative anterior resection of rectum, with extraperitoneal anastomosis (of the rectum) <10cm from the anal verge, with or without covering stoma |
| 32026 | Ultra low restorative resection of rectum, with or without covering stoma, where the anastomosis is sited in the anorectal region and is 6cm or less from the anal verge |
| 32028 | Low or ultra low restorative resection of rectum, with peranal sutured coloanal anastomosis, with or without covering stoma |
| 32030 | Rectosigmoidectomy (Hartmann’s operation) |
| 32033 | Restoration of bowel following Hartmann's or similar operation, including dismantling of the stoma |
| 32039 | Abdominoperineal resection of rectum and anus, 1 surgeon |
| 32047 | Perineal proctectomy |
| 32051 | Total colectomy with excision of rectum and ileoanal anastomosis with formation of ileal reservoir, with or without creation of temporary ileostomy, 1 surgeon/2 surgeons |
| 32099 | Per anal submucosal excision of rectal tumour of 5cm or less in diameter |
| 32105 | Per anal full thickness excision of anorectal carcinoma |
| 32108 | Transsphincteric excision of rectal tumour (Kraske or similar operation) |
| 32112 | Perineal recto-sigmoidectomy for rectal prolapse |

Table S6. MBS item codes to identify radiotherapy for colorectal cancer.

| **Code** | **Description** |
| --- | --- |
| 15215- 15242 | Radiation oncology treatment, using a single photon energy linear accelerator with or without electron facilities, delivered to primary / secondary site, each attendance at which treatment is given, 1 field / 2+ fields up to a maximum of 5 fields |
| 15245-15272 | Radiation oncology treatment, using a dual photon energy linear accelerator with a minimum higher energy of at least 10mv photons, with electron facilities, delivered to primary / secondary site, each attendance at which treatment is given, 1 field / 2+ fields up to a maximum of 5 fields |
| 15500 / 15503 / 15506 | Radiation field setting using simulator or isocentric xray or megavoltage machine or CT of: a single area for treatment by a single field or parallel opposed fields / a single area where views in >1 plane are required for treatment by multiple fields, or of 2 areas / 3+ areas or of total or half body irradiation, or of mantle therapy or inverted Y fields, or of irregularly shaped fields using multiple blocks, or of offaxis fields or several joined fields |
| 15509 / 15512 / 15515 | Radiation field setting using diagnostic x-ray unit of: a single area for treatment by a single field or parallel opposed fields / a single area where views in >1 plane are required for treatment by multiple fields, or of 2 areas / 3+ areas or of total or half body irradiation etc (as per previous group) |
| 15518 / 15521 / 15524 | Radiation dosimetry by a CT interfacing planning computer for megavoltage or teletherapy radiotherapy: by a single field or parallel opposed fields to 1 area with up to 2 shielding blocks / to a single area by 3+ fields or by a single field or parallel opposed fields to 2 areas or where wedges are used / to 3+ areas, or by mantle fields or inverted Y fields or tangential fields or irregularly shaped fields using multiple blocks, or offaxis fields, or several joined fields |
| 15527 / 15530 / 15533 | Radiation dosimetry by a non CT interfacing planning computer for megavoltage or teletherapy radiotherapy: by a single field or parallel opposed fields to 1 area with up to 2 shielding blocks / to a single area by 3+ fields or by a single field or parallel opposed fields to 2 areas or where wedges are used / to 3+ areas, or by mantle fields or inverted Y fields etc (as per previous group) |
| 15550 / 15553 | Simulation for 3D conformal radiotherapy: without intravenous contrast medium / pre and post intravenous contrast medium |
| 15556-15562 | Dosimetry for 3D conformal radiotherapy of complexity level 1/2/3 |
| 15600 | Stereotactic radiosurgery including all radiation oncology consultations, planning, simulation, dosimetry, treatment |
| 15700-15710 | Radiation oncology treatment verification – single projection / multiple projection / volumetric acquisition |

Table S7. MBS item codes to identify chemotherapy for colorectal cancer.

| **Code** | **Description** |
| --- | --- |
| 13915 | Cytotoxic chemotherapy, administration of, by intravenous infusion <= 1 hours duration |
| 13918 | Cytotoxic chemotherapy, administration of, by intravenous infusion >1 - 6 hours duration |
| 13921 | Cytotoxic chemotherapy, administration of, by intravenous infusion >6 hours - for the first day of treatment |
| 13924 | Cytotoxic chemotherapy, administration of, by intravenous infusion >6 hours duration - on each day subsequent to the first in the same continuous treatment episode |
| 13927 | Cytotoxic chemotherapy, administration of, by intra-arterial administration <= 1 hours duration |
| 13930 | Cytotoxic chemotherapy, administration of, by intra-arterial infusion >1 - 6 hours duration |
| 13933 | Cytotoxic chemotherapy, administration of, by intra-arterial infusion >6 hours - for the first day of treatment |
| 13936 | Cytotoxic chemotherapy, administration of, by intra-arterial infusion >6 hours duration - on each day subsequent to the first in the same continuous treatment episode |
| 13945 | Accessing long-term implanted drug delivery device for cytotoxic chemotherapy |

Table S8. PBS items to identify chemotherapy for colorectal cancer.

| **Code** | **Description** |
| --- | --- |
| (various) | Fluororacil |
| (various) | Capecitabine |
| (various) | Cetuximab |
| (various) | Irinotecan |
| (various) | Irinotecan hydrochloride trihydrate |
| (various) | Oxaliplatin |

**Codes to potentially identify lung cancers**

Table S9. APDC procedure codes to identify surgical resection for lung cancer.

| **Code** | **Description** |
| --- | --- |
| 38438-00 | Segmental resection of lung |
| 38438-01 | Lobectomy of lung |
| 38438-02 | Pneumonectomy |
| 38440-00 | Wedge resection of lung |
| 38440-01 | Radical wedge resection of lung |
| 38441-00 | Radical lobectomy |
| 38441-01 | Radical pneumonectomy |
| 90169-00 | Endoscopic wedge resection of lung |

Table S10. APDC procedure codes and diagnosis codes to identify chemotherapy for lung cancer.

| **Data type** | **Code** | **Description** |
| --- | --- | --- |
| Procedure | 13915-00 | Chemotherapy, intravenous administration <= 1 hours duration |
| Procedure | 13918-00 | Chemotherapy, intravenous administration >1 - 6 hours duration |
| Procedure | 13921-00 | Chemotherapy, intravenous administration >6 hours duration |
| Procedure | 90760-00 | Chemotherapy, oral administration |
| Procedure | 90767-00 | Chemotherapy, subcutaneous or intramuscular administration |
| Procedure | 90768-00 | Chemotherapy, other administration |
| Diagnosis | Z51.1 | Pharmacotherapy session for neoplasm |
| Diagnosis | Z51.2 | Other chemotherapy |

Table S11. APDC procedure codes and diagnosis codes to identify radiotherapy for lung cancer.

| **Data type** | **Code** | **Description** |
| --- | --- | --- |
| Procedure | 15203-00 | Radiation treatment, megavoltage, single photon energy machine, 1 field |
| Procedure | 15204-00 | Radiation treatment, megavoltage, single photon energy machine, >= 2 fields |
| Procedure | 15207-00 | Radiation treatment, megavoltage, dual photon energy machine, 1 field |
| Procedure | 15208-00 | Radiation treatment, megavoltage, dual photon energy machine, >= 2 fields |
| Procedure | 15224-00 | Radiation treatment, megavoltage, single modality linear accelerator, 1 field |
| Procedure | 15239-00 | Radiation treatment, megavoltage, single modality linear accelerator, >= 2 fields |
| Procedure | 15254-00 | Radiation treatment, megavoltage, dual modality linear accelerator, 1 field |
| Procedure | 15269-00 | Radiation treatment, megavoltage, dual modality linear accelerator, >= 2 fields |
| Procedure | 15506-01 | Radiation field setting using dedicated CT scanner |
| Procedure | 15506-02 | Radiation field setting using intensity modulated radiation therapy |
| Procedure | 15521-00 | Dosimetry by CT interfacing computer, intermediate |
| Procedure | 15550-00 | Radiation field setting for three dimensional conformal radiation therapy |
| Procedure | 15600-00 | Stereotactic radiation treatment, single dose |
| Procedure | 15600-01 | Stereotactic radiation treatment, fractionated |
| Procedure | 90765-00 | Construction and fitting of immobilisation device, simple |
| Procedure | 90765-01 | Construction and fitting of immobilisation device, intermediate |
| Procedure | 90765-02 | Construction and fitting of immobilisation device, complex |
| Procedure | 90765-03 | Construction and fitting of customised blocks |
| Diagnosis | Z51.0 | Radiotherapy session |

Table S12. EDDC diagnosis codes to identify lung cancer.

| **Classification system** | **Code** | **Description** |
| --- | --- | --- |
| ICD10-AM | C34 | Malignant neoplasm of bronchus and lung |
| ICD9 | 162.9 | Malignant neoplasm of trachea, bronchus and lung |
| SNOMED | 93880001 | Primary malignant neoplasm of lung (disorder) |
| SNOMED | 126712008 | Neoplasm of bronchus (disorder) |
| SNOMED | 126713003 | Neoplasm of lung (disorder) |

Table S13. MBS item codes to identify surgical resection for lung cancer.

| **Code** | **Description** |
| --- | --- |
| 38438 | Pneumonectomy or lobectomy or segmentectomy |
| 38440 | Wedge resection of lung |
| 38441 | Radical lobectomy or pneumonectomy including resection of chest wall, diaphragm, pericardium, or formal mediastinal node dissection |

Table S14. MBS item codes to identify radiotherapy for lung cancer.

| **Code** | **Description** |
| --- | --- |
| 15215- 15242 | Radiation oncology treatment, using a single photon energy linear accelerator with or without electron facilities, delivered to primary / secondary site, each attendance at which treatment is given, 1 field / 2+ fields up to a maximum of 5 fields |
| 15245-15272 | Radiation oncology treatment, using a dual photon energy linear accelerator with a minimum higher energy of at least 10mv photons, with electron facilities, delivered to primary / secondary site, each attendance at which treatment is given, 1 field / 2+ fields up to a maximum of 5 fields |
| 15500 / 15503 / 15506 | Radiation field setting using simulator or isocentric xray or megavoltage machine or CT of: a single area for treatment by a single field or parallel opposed fields / a single area where views in >1 plane are required for treatment by multiple fields, or of 2 areas / 3+ areas or of total or half body irradiation, or of mantle therapy or inverted Y fields, or of irregularly shaped fields using multiple blocks, or of offaxis fields or several joined fields |
| 15509 / 15512 / 15515 | Radiation field setting using diagnostic x-ray unit of: a single area for treatment by a single field or parallel opposed fields / a single area where views in >1 plane are required for treatment by multiple fields, or of 2 areas / 3+ areas or of total or half body irradiation etc (as per previous group) |
| 15518 / 15521 / 15524 | Radiation dosimetry by a CT interfacing planning computer for megavoltage or teletherapy radiotherapy: by a single field or parallel opposed fields to 1 area with up to 2 shielding blocks / to a single area by 3+ fields or by a single field or parallel opposed fields to 2 areas or where wedges are used / to 3+ areas, or by mantle fields or inverted Y fields or tangential fields or irregularly shaped fields using multiple blocks, or offaxis fields, or several joined fields |
| 15527 / 15530 / 15533 | Radiation dosimetry by a non CT interfacing planning computer for megavoltage or teletherapy radiotherapy: by a single field or parallel opposed fields to 1 area with up to 2 shielding blocks / to a single area by 3+ fields or by a single field or parallel opposed fields to 2 areas or where wedges are used / to 3+ areas, or by mantle fields or inverted Y fields etc (as per previous group) |
| 15550 / 15553 | Simulation for 3D conformal radiotherapy: without intravenous contrast medium / pre and post intravenous contrast medium |
| 15556-15562 | Dosimetry for 3D conformal radiotherapy of complexity level 1/2/3 |
| 15600 | Stereotactic radiosurgery including all radiation oncology consultations, planning, simulation, dosimetry, treatment |
| 15700-15710 | Radiation oncology treatment verification – single projection / multiple projection / volumetric acquisition |

Table S15. MBS item codes to identify chemotherapy for lung cancer.

| **Code** | **Description** |
| --- | --- |
| 13915 | Cytotoxic chemotherapy, administration of, by intravenous infusion <= 1 hours duration |
| 13918 | Cytotoxic chemotherapy, administration of, by intravenous infusion >1 - 6 hours duration |
| 13921 | Cytotoxic chemotherapy, administration of, by intravenous infusion >6 hours - for the first day of treatment |
| 13924 | Cytotoxic chemotherapy, administration of, by intravenous infusion >6 hours duration - on each day subsequent to the first in the same continuous treatment episode |
| 13927 | Cytotoxic chemotherapy, administration of, by intra-arterial administration <= 1 hours duration |
| 13930 | Cytotoxic chemotherapy, administration of, by intra-arterial infusion >1 - 6 hours duration |
| 13933 | Cytotoxic chemotherapy, administration of, by intra-arterial infusion >6 hours - for the first day of treatment |
| 13936 | Cytotoxic chemotherapy, administration of, by intra-arterial infusion >6 hours duration - on each day subsequent to the first in the same continuous treatment episode |
| 13945 | Accessing long-term implanted drug delivery device for cytotoxic chemotherapy |

Table S16. PBS items to identify chemotherapy for lung cancer.

| **Code** | **Description** |
| --- | --- |
| (various) | Cisplatin |
| (various) | Carboplatin |
| (various) | Docetaxel |
| (various) | Erlotinib |
| (various) | Etanercept |
| (various) | Gefitinib |
| (various) | Gemcitabine |
| (various) | Gemcitabine hydrochloride |
| (various) | Paclitaxel |
| (various) | Paclitaxel nanoparticle |
| (various) | Vinorelbine |
| (various) | Vinorelbine tartrate |
| (various) | Pemetrexed |
| (various) | Pemetrexed disodium |
